# Case Reports of a New Method for Differential Diagnosis of Calcified Carotid Artery Atheroma

**DOI:** 10.1155/2021/8874087

**Published:** 2021-01-04

**Authors:** Guilherme Augusto Alves de Oliveira, Cleiterson Rezende de Sá, Omar Ribeiro Santos Junior, Rafael Pereira da Mata Santos, Flávio Ricardo Manzi

**Affiliations:** ^1^Department of Dentistry of the Pontifical Catholic University of Minas Gerais (PUC-MG), Belo Horizonte, Minas Gerais, Brazil; ^2^Preventive Cardiology Department of Núcleo Cardiológico Integrado Ltda., Mateus Leme, Minas Gerais, Brazil

## Abstract

*Introduction*. Early diagnosis of calcified atheromas may decrease morbidity and mortality caused by brain and cardiovascular diseases, in which atherosclerosis is the main etiological factor of these pathologies. Dental examinations with the aim of detecting this pathology have been in progress since 1981, such as panoramic radiography, considered the most widely studied method for this diagnosis. However, some limitations of this exam have been reported with reference to inability to visualize the cervical region and difficulty of establishing a precise diagnosis because of many structures and calcifications that have similar radiographic characteristics. *Case Report*. The present study to describe a dental radiographic technique for establishing the differential diagnosis of calcified atheromas regarding other calcifications and reporting 3 clinical cases that demonstrate its effectiveness in different clinical situations. *Discussion*. Manzi Projection can promote a differential diagnosis of calcified atheromas in dental practice and consequently subsidize the clinician for referring the patient to the physician.

## 1. Introduction

Atherosclerosis may be considered the main cause of the global death rate due to its association with the etiology of cardiovascular and cerebrovascular diseases. Clinically, it is characterized by the formation of atheromatous plaques between the tunica intima and tunica media of the arterial wall [1]. The bifurcation region of the carotid artery represents one of the main sites for the development of atheroma, favored by the presence of a turbulent flow that generates a low intensity yet constant stress on the arterial wall, causing endothelial lesions that will trigger the formation of these lipoprotein plates [2].

These atheromas may undergo a dystrophic calcification process, enabling these plates to be identified in radiographic examinations, e.g., the panoramic radiograph. In this examination, widely used in dental practice, calcified atheroma may be visualized as circled, irregular or heterogeneous, unilateral or bilateral radiopaque masses in the neck soft tissue region, adjacent to the C3-C4 intervertebral space, distinct from the radiopaque structures of this region, next to the hyoid bone [3, 4].

Many other calcifications and structures represented in the cervical region of the panoramic radiograph may make it difficult to diagnose calcified atheromas, as they may present similar radiographic characteristics, such as triticeous cartilage calcification, sialoliths, phleboliths, hyoid bone, calcified lymph nodes, and among others. Thus, complementary examinations are necessary for establishing a differential diagnosis [5, 6].

Notably, Intra-arterial Angiography is the gold standard for the detection of these atheromatous plaques and the stenosis they cause; however, because it is an invasive method with an estimated risk morbidity and mortality of 2%, noninvasive examinations are chosen in clinical practice, in which the Doppler Ultrasound is outstanding, as it has an accuracy rate of up to 90% when compared with Intra-arterial Angiography [7–9].

The purpose of this study is to describe a dental radiographic technique for establishing the differential diagnosis of calcified atheromas relative to other calcifications with similar radiographic characteristics. Furthermore, the intention is to report three clinical cases that demonstrate the diagnostic possibilities of this technique.

## 2. Technical Report

**Figure 1:**
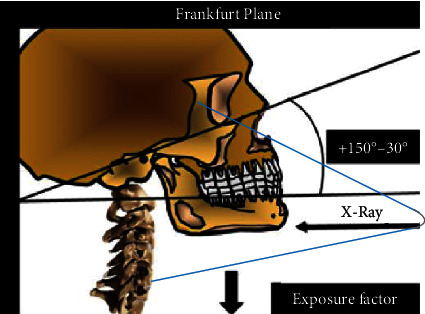
Manzi Projection description.

The Manzi Projection is performed using the cephalometric unit of the panoramic radiographic equipment, in which the technique is described as follows: anteroposterior incidence, Frankfurt plane with vertical inclination between +15 and 30°, angle of the vertical and horizontal X-ray central beam at 0°, and reduction of radiation exposure factors, usually at 50% (Figure 1).

## 3. Case Reports

### 3.1. Clinical Case 1

**Figure 2:**
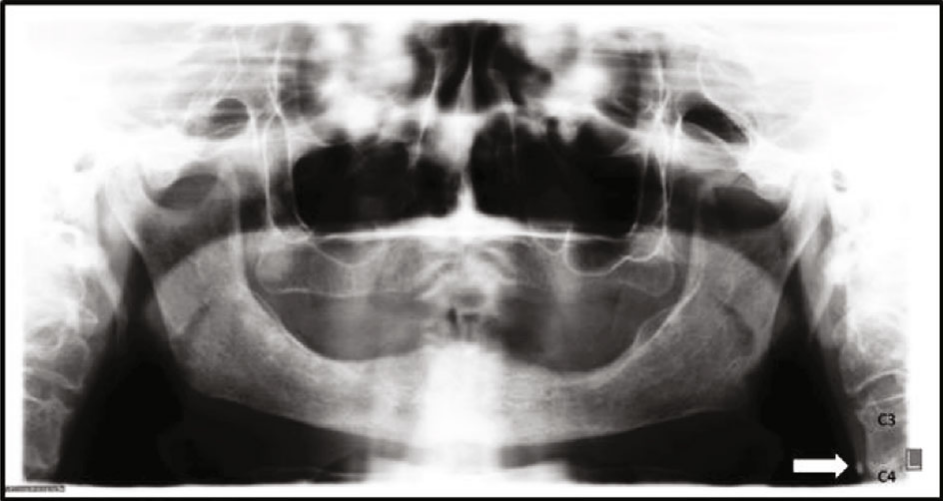
Panoramic radiograph showing radiopaque image circumscribed on the left side of the neck soft tissue region adjacent to the C3 and C4 intervertebral space (arrow).

The patient, a 78-year-old woman, systemically presenting the following risk factors related to atheroma formation: diabetes, dyslipidemia, arterial hypertension, and tobacco use. The patient was asked to have a panoramic radiograph taken for dental treatment. The examination was performed with the Kodak Ceph 9000 3D (Carestream Health, Inc.) equipment, in which the presence of radiopaque mass adjacent to the C3-C4 intervertebral space on the left side of the patient was detected, compatible with calcified atheroma (Figure 2).

**Figure 3:**
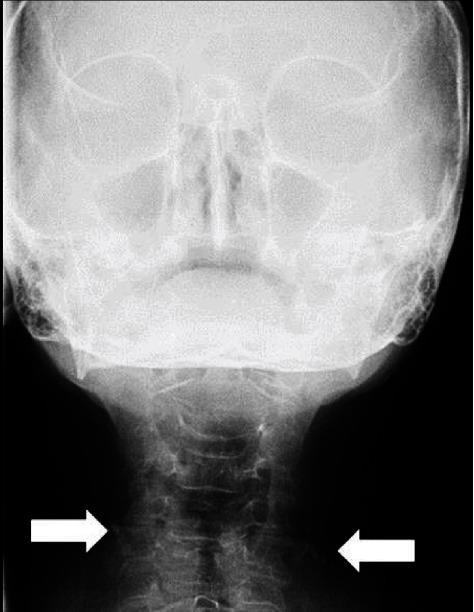
Manzi Projection showing radiopaque image circumscribed on both sides of the neck soft tissue region, in which the left side is adjacent to the C3 and C4 intervertebral space (arrow) and the right side is adjacent to the C4 (arrow).

The Manzi Projection was then performed on the patient to establish a differential diagnosis between calcified atheromas and other calcifications with similar imaginological characteristics. This was performed with the cephalometric unit of the Kodak Ceph 9000 3D (Carestream Health, Inc., Rochester, NY, USA) equipment, by which the presence of bilateral radiopaque masses were detected in the patient, showing that the one on the right side was located adjacent to the C4 vertebra (Figure 3).

As these calcifications were also presented in the Manzi Projection, the patient was referred for cardiac monitoring, in which the Doppler Ultrasound was requested to establish the degree of arterial stenosis. The presence of calcified plaque in the carotid bulb (bulbar or medulla oblongata) region of the right and left carotid arteries was detected in this examination, causing between 30 and 49% stenosis.

### 3.2. Clinical Case 2

**Figure 4:**
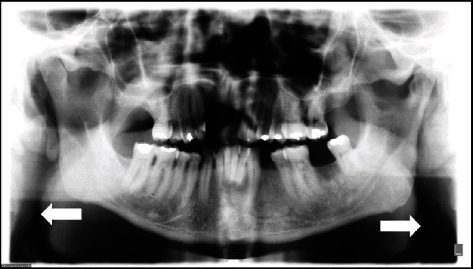
Panoramic radiograph showing the presence of bilateral radiopaque masses (arrows).

The patient, a 56-year-old man, systemically presenting the following risk factors for atherosclerosis: dyslipidemia, arterial hypertension, active periodontal disease, and being a self-declared ex-tobacco user. The panoramic radiograph for dental treatment was requested, in which the presence of bilateral radiopaque masses adjacent to the cervical region was detected (Figure 4).

**Figure 5:**
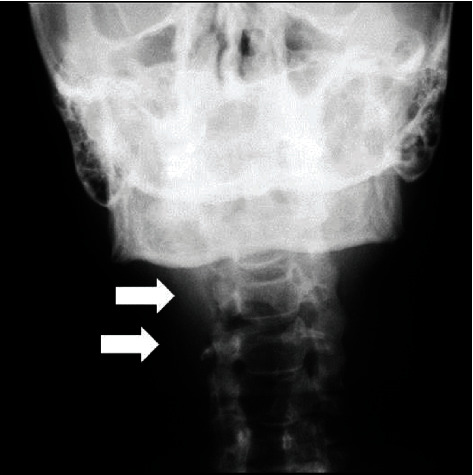
Manzi Projection showing the presence of two calcified plaques adjacent to the cervical region (arrows).

The patient was asked to undergo the Manzi Projection examination, which demonstrated the presence of radiopaque masses compatible with calcified atheromas on the patient's right side, in which the presence of a second plaque, in a more inferior position than that of the one demonstrated by the panoramic radiograph, and the absence of calcified plaque on the left of the patient were verified (Figure 5).

The patient was referred to the responsible cardiologist, who confirmed, by Doppler Ultrasound, the presence of calcified plaques in the right carotid artery, located in the carotid bulb and common carotid artery region, causing between 30 and 49% stenosis. On the left side, the presence of calcium free plaque was also verified, causing between 30 and 49% stenosis. However, the calcification visualized on the left side of the panoramic radiograph was not a calcified atheroma.

### 3.3. Clinical Case 3

**Figure 6:**
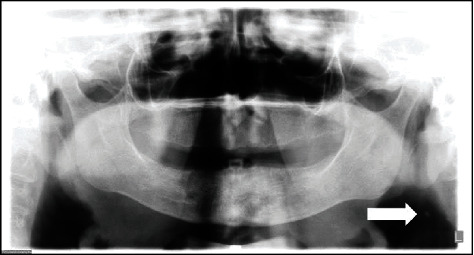
Panoramic radiograph showing the presence of radiopaque mass circumscribed on the left side of the cervical region (arrow).

The patient, a 90-year-old woman, systemically presenting the following risk factors for atherosclerosis: dyslipidemia and arterial hypertension. She was submitted to panoramic radiograph examination for dental treatment in which the presence of a calcified plaque was verified and visualized in the cervical region on the patient's left side (Figure 6).

**Figure 7:**
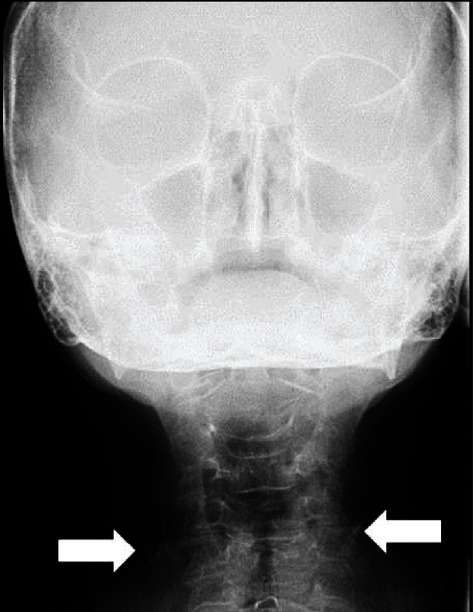
Manzi Projection showing the presence of bilateral radiopaque masses, adjacent to the cervical region (arrows). Notice that these structures are inferior to the calcified structure region demonstrated by the panoramic radiograph.

The Manzi Projection for the differential diagnosis of other calcifications and structures was performed in the dental office, in which the presence of radiopaque masses adjacent to the cervical region was verified, on both sides (bilateral). Relevant to highlight is that the localization of the calcified structure (demonstrated by the panoramic radiograph) was superior position to that of the structures visualized on the Manzi Projection and that these structures did not appear to be prominently in the cervical region, the region of the carotid artery itself (Figure 7).

The patient was referred for cardiac monitoring, in which the Doppler Ultrasound was requested. Thus, on examination, the presence of bilateral calcified plaques in the carotid bulb and common carotid artery regions was verified, causing over 70% stenosis on the right side and between 50 and 69% stenosis on the left side.

## 4. Discussion

Atherosclerosis is usually asymptomatic, and it is diagnosed by means of medical examinations, such as Doppler Ultrasound, considered the most widely used examination for this purpose, because it is a noninvasive method with excellent accuracy. It differs from Intra-arterial Angiography which, although highly accurate, is an invasive method with an estimated rate of morbidity and mortality of 2% [7–9]

Other calcifications in the soft tissues can be observed radiographically in the cervical region. Carotid atheroma must be distinguished from those other radiopacities that lie in proximity to the artery. Based on the location, the calcified triticeous cartilage is the most likely calcification to be confused with a carotid atheroma. However, as a matter of distinguishing, there are some main features that can be observed. Calcified triticeous cartilages are well delimited and regular radiopacities, while carotid atheroma is visualized as irregular, heterogeneous, verticolinear radiopacities. Also, in a panoramic radiograph, the carotid atheroma appears more laterally than a calcified triticeous cartilage [10, 11].

Intending to provide an early diagnosis of these calcified atheromatous plaques, Friedlander and Lande, as far back as 1981, analyzed the possibility of identifying these calcifications by means of using panoramic radiography. Thirty-five years have passed, and this method is still being studied and compared with other medical examinations for establishing its sensitivity, specificity, and accuracy. Although the panoramic radiograph does not present similar rates when compared with Doppler Ultrasound, its findings must not be disregarded, as the literature infers an association between the presence of calcified plaques in the cervical region (panoramic) and carotid calcifications [10–13].

The Manzi Projection represents a dental radiographic technique that may assist with the differential diagnosis of these calcified atheromatous plaques in the carotid because, in this examination, the course of the carotid artery is displayed without being superimposed by other calcified structures, and enlargement of the vertebral visualization enables an analysis of the cervical region. This is important because around 11% of the carotid bifurcations, the sites most frequently affected by atheromatous plaques are found inferior to the C3-C4 intervertebral space, where visualization in the panoramic radiograph is most unlikely [14]. The patient undergoes the examination with the Frankfurt plane (that starts at the superior portion of the acoustic meatus and is tangential to the infraorbital foramen) leaning slightly in the superior direction (between 15 and 30°), which diminishes the possibility of mandibular superimposition of the cervical region. Because it is an anteroposterior technique, the region of the carotid bifurcation is found closer to the radiographic sensor which, according to the geometric principles of radiological image formation, may clearly distinguish calcified atheromas, in case they exist.

## 5. Conclusion

The Manzi Projection can promote differential diagnosis of calcified atheroma in relation to other cervical calcifications in dental practice; however, further studies are necessary to establish its accuracy.

## Additional Points

*Human and Animal Rights*. No animals were used in this research. All research procedures followed were in accordance with the ethical standards of the committee responsible for human experimentation (institutional and national) and with the Helsinki Declaration of 1975, as revised in 2008 (http://www.wma.net/en/20activities/10ethics/10helsinki/).
